# High Stroke Volume Variation Method by Mannitol Administration Can Decrease Blood Loss During Donor Hepatectomy

**DOI:** 10.1097/MD.0000000000002328

**Published:** 2016-01-15

**Authors:** Hyungseok Seo, In-Gu Jun, Tae-Yong Ha, Shin Hwang, Sung-Gyu Lee, Young-Kug Kim

**Affiliations:** From the Department of Anesthesiology and Pain Medicine, Seoul National University Hospital (HS); Department of Anesthesiology and Pain Medicine, Asan Medical Center, University of Ulsan College of Medicine (I-GJ, Y-KK); and Division of Liver Transplantation and Hepatobiliary Surgery, Department of Surgery, Asan Medical Center, University of Ulsan College of Medicine, Seoul, Republic of Korea (T-YH, SH, S-GL).

## Abstract

Optimal fluid management to reduce blood loss during donor hepatectomy is important for maximizing donor safety. Mannitol can induce osmotic diuresis, helping prevent increased intravascular volume status. We therefore evaluated the effect of high stroke volume variation (SVV) method by mannitol administration and fluid restriction on blood loss during donor hepatectomy.

In this prospective study, 64 donors scheduled for donor right hepatectomy were included and allocated into 2 groups. In group A, the SVV value of each patient was maintained at 10% to 20% during hepatic resection with 0.5 g/kg mannitol administration and fluid restriction at a rate of 2 to 4 mL/kg/h. In group B, the SVV value was maintained at <10% by fluid administration at a rate of 6 to 10 mL/kg/h without diuretic administration during surgery. Intraoperative blood loss was estimated by the loss of red cell mass. Surgeon satisfaction scores and postoperative outcomes, including acute kidney injury, abnormal chest radiographic findings, and hospital stay duration, were also assessed.

SVV during hepatectomy was significantly higher in group A than in group B (11.0 ± 1.7 vs 6.5 ± 1.1, *P* < 0.001). The red cell mass loss was significantly lower in group A than in group B (145.4 ± 107.6 vs 307.9 ± 110.7 mL, *P* < 0.001). Surgeon satisfaction scores were higher in group A than in group B (2.8 ± 0.5 vs 2.0 ± 0.6, *P* < 0.001). The incidence of acute kidney injury, abnormal chest radiographic findings, and duration of hospital stay did not significantly differ between the 2 groups.

Maintenance of high SVV by mannitol administration is effective and safe for reducing blood loss during donor hepatectomy.

## INTRODUCTION

Donor safety during hepatectomy is a crucial issue in adult living donor liver transplantation. Bleeding is an anticipated complication, particularly in hepatic surgery, and subsequent blood transfusion can increase postoperative morbidity and mortality.^1–3^ Therefore, optimal fluid management to reduce intraoperative bleeding and subsequent blood transfusion is important.

Stroke volume variation (SVV), which is used as a dynamic volume index, can be useful for evaluating intravascular volume status^4–6^ and replacing conventional central venous pressure (CVP) monitoring during hepatic resection.^7^ We found that a high SVV method by furosemide administration and fluid restriction reduced blood loss during donor hepatectomy.^8^ However, furosemide may be associated with renal injury in many clinical situations.^9–11^ Therefore, alternative fluid management protocols for safely and effectively maintaining a high SVV are required to maximize the safety of living liver donors.

Mannitol, an osmotic diuretic, can reduce vascular congestion within a surgical field. Furthermore, it acts as a free radical scavenger^12,13^ and protects against ischemia-reperfusion injury.^14,15^ Therefore, we compared the effects of high SVV (10–20%), maintained by mannitol administration and fluid restriction, and low SVV (<10%), maintained by a conventional fluid management protocol without mannitol administration, on blood loss during donor hepatectomy. We also compared postoperative outcomes, including acute kidney injury, liver dysfunction, and abnormal chest radiographic findings, between the 2 fluid management protocols.

## METHODS

### Patients

This prospective study was conducted between July 2014 and November 2014. The study protocol was approved by the Asan Medical Center Institutional Review Board (approval number: 2014-0625) and registered on the international clinical trials registry platform (http://cris.nih.go.kr; KCT0001157). A total of 64 donors scheduled for right hepatectomy for living donor liver transplantation were included and allocated into 2 groups. In group A, the SVV value of each patient was maintained at 10% to 20% during the hepatic resection period, whereas in group B, the SVV value was maintained at <10%. Written informed consent was obtained from all patients. Donors younger than 20 years of age or with cardiac arrhythmia were excluded.

### Anesthesia Protocol

Anesthesia management for living donor hepatectomy was performed in accordance with our institutional standards.^16,17^ Briefly, anesthesia was induced with 5 mg/kg thiopental sodium and 1 to 2 μg/kg fentanyl, and 0.6 mg/kg rocuronium was used to facilitate tracheal intubation. Anesthesia was maintained at 1 to 2% sevoflurane in addition to the intermittent bolus administration of fentanyl and rocuronium. Fresh gas flow was maintained at 2.0 L/min with an inspired oxygen fraction of 0.5 in an oxygen/nitrous oxide mixture. Mechanical ventilation was performed using a tidal volume of 8 to 10 mL/kg and a respiratory rate of 10 to 12/min. Arterial carbon dioxide tension was maintained within 35 to 40 mm Hg. The depth of anesthesia was monitored using the bispectral index (BIS, A-1050 Monitor, Aspect Medical Systems, Newton, MA), which was maintained at 40 to 60. Direct arterial blood pressure was monitored using a radial artery catheter. After zeroing against atmosphere, an indwelling radial arterial catheter was simultaneously connected to an EV1000 (Edwards Lifesciences LLC, Irvine, CA) for SVV monitoring. A 3-lumen central venous catheter was inserted into the right internal jugular vein.

Fluid management varied between groups A and B. In group A, in order to maintain an SVV of 10% to 20% from the end of anesthesia induction to the completion of hepatic resection, 0.5 g/kg mannitol was routinely administered for 20 min after anesthesia induction and a crystalloid solution (PlasmaLyte, Baxter Healthcare Corporation, Deerfield, IL) was administered at a rate of 2 to 4 mL/kg/h. From the completion of hepatic resection to the end of surgery, the crystalloid solution was administered at a rate of 8 to 10 mL/kg/h. In group B, in order to maintain an SVV of <10% from the end of anesthesia induction to the completion of hepatic resection, only the crystalloid solution was administered at a rate of 6 to 10 mL/kg/h, and no diuretics were used. From the completion of hepatic resection to the end of surgery, the crystalloid solution continued to be administered at a rate of 6 to 10 mL/kg/h. In both groups, a colloid solution (20% albumin) was administered from the completion of hepatic resection to the end of surgery for volume replacement. Systolic arterial blood pressure (SAP) was maintained at ≥90 mm Hg in both groups. A hypotensive episode was defined as SAP <90 mm Hg during hepatic resection. If SAP was < 80 mm Hg during hepatectomy, additional fluid and ephedrine or phenylephrine was administered. Urine output was maintained at ≥0.5 mL/kg/h. Intraoperative red blood cell (RBC) transfusion was indicated when the serum hemoglobin level was <7.0 g/dL.

### Outcome Measurements

Intraoperative blood loss was estimated using loss of red cell mass, which was derived from differences in pre- and postoperative hematocrits and transfused red cell mass with the following equation:^18^ loss of red cell mass (mL) = estimated blood volume of patient (mL) × (preoperative hematocrit (%) − immediate postoperative hematocrit (%))/100 + (transfused packed RBCs [unit] × 213 × 0.7) (estimated blood volume of patient [mL] = 75 mL/kg for men or 65 mL/kg for women × body weight [kg]; 213 mL for average volume of packed RBC; 0.7 value for hematocrit of packed RBCs).

Hemodynamic parameters, including SVV, mean arterial blood pressure (MAP), heart rate, CVP, cardiac output, and systemic vascular resistance, were measured at 5 specific time points; 15 min after anesthesia induction (T1), just before hepatic resection (T2), during hepatic resection (T3), just after the completion of hepatic resection (T4), and at the end of surgery (T5). At each time point, arterial blood gas and serum electrolyte levels were evaluated. For intraoperative variables, the incidence of intraoperative hypotension, amount of administered vasopressor, and amount of administered fluid were assessed. After the completion of the entire surgical procedure, all surgeons were asked for their overall satisfaction score for the surgical field on a 5-point scale as follows: excellent (4), good (3), fair (2), poor (1), and extremely poor (0).

Laboratory tests, including hematocrit, liver function tests, and serum creatinine, were used to evaluate postoperative outcomes. Postoperative acute kidney injury was diagnosed if any one of the following was present: increase in the serum creatinine level by ≥0.3 mg/dL within 48 h; increase in the serum creatinine level to ≥1.5 times baseline within 7 days; or urine volume <0.5 mL/kg/h for 6 h. Newly developed abnormal findings on chest radiography (ie, atelectasis, pleural effusion, or pulmonary congestion) and hospital stay lengths were also evaluated.

### Statistics

In our previous study, the blood loss during donor hepatectomy was measured at 691 ± 365.5 mL.^16^ We assumed a 40% difference in the intraoperative blood loss between the 2 fluid management protocols. Considering a type I error of 0.05 and a desired power of 0.80, 29 patients in each group were required for our present analysis. Assuming a 10% dropout rate, 32 patients were included in each group. All data sets were included in the modified intention-to-treat analysis. Changes in variables measured at each time point were assessed using repeated measures analysis of variance, and inter-group differences were compared using Student's *t* test. Categorical data between the 2 groups were compared using the chi-square test or Fisher's exact test. All results are expressed as mean ± SD or number (percentage). Statistical analysis was performed using MedCalc^®^ version 13.2.0 (MedCalc software, Ostend, Belgium) or SigmaPlot 10.0 (Systat Software, Inc, San Jose, CA). A *P* value < 0.05 was considered statistically significant.

## RESULTS

Sixty-four donors who underwent a right hepatectomy for living donor liver transplantation were included and allocated into either group A (SVV maintained at 10–20% during hepatectomy) or group B (SVV maintained <10% during hepatectomy). In group A, we failed to maintain the target SVV levels in 6 donors, despite mannitol administration and fluid restriction. However, these 6 donors were still included in the final modified intention-to-treat analysis to reduce bias.

Donor characteristics and intraoperative data are presented in Table 1. There were no significant differences in demographic variables between group A and group B. In group A, the amount of administered crystalloid was significantly smaller, whereas the urine output was significantly greater. SVV during hepatectomy was significantly higher in group A than in group B (11.0 ± 1.7 vs 6.5 ± 1.1%, *P* < 0.001), whereas CVP during hepatectomy was significantly lower in group A than in group B (3.3 ± 1.0 vs 5.5 ± 1.3 mm Hg, *P* < 0.001). However, other hemodynamic parameters, including cardiac output, systemic vascular resistance, MAP, and heart rate, were not significantly different between the 2 groups at any time point. Group A had a higher surgeon satisfaction score than group B (2.8 ± 0.5 vs 2.0 ± 0.6, *P* < 0.001).

**TABLE 1:**
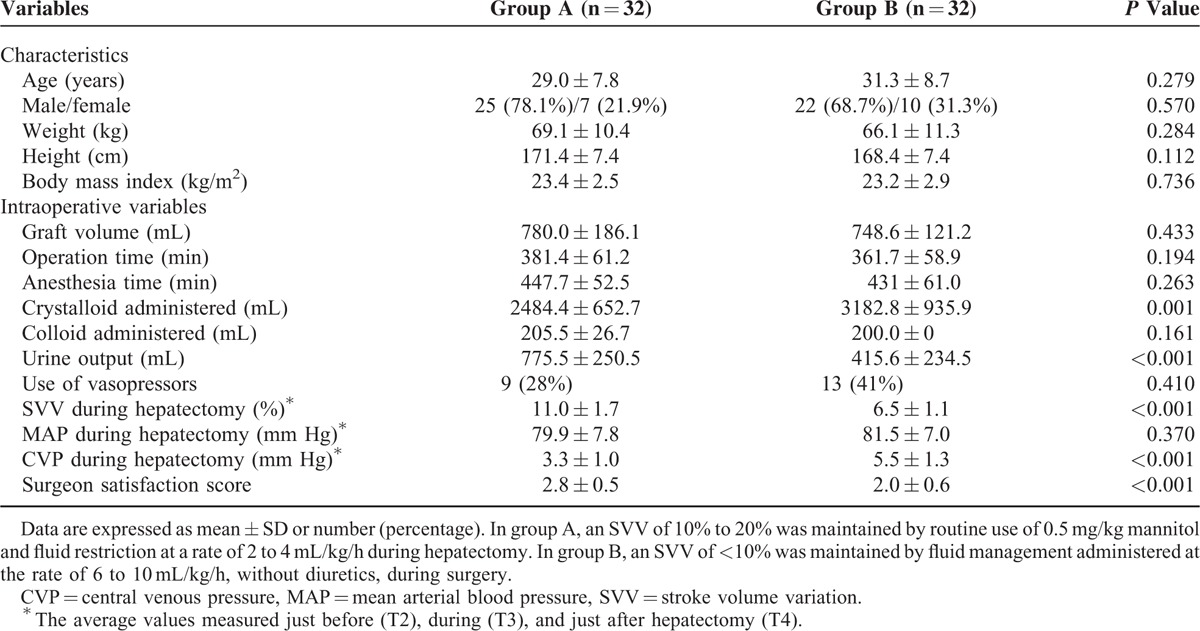
Donor Characteristics and Intraoperative Variables

**FIGURE 1:**
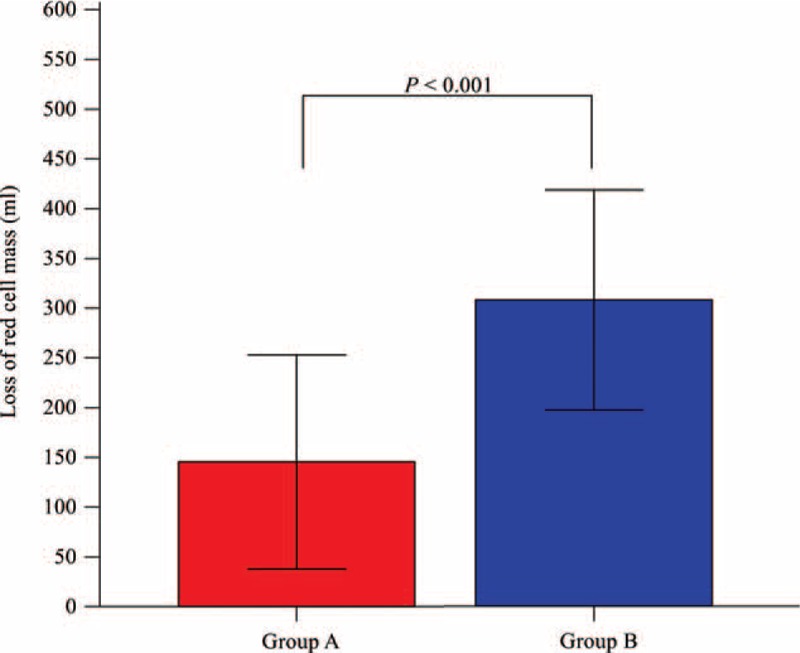
Loss of red cell mass during donor hepatectomy in groups A and B. In group A (red box), an SVV of 10% to 20% was maintained by routine use of 0.5 mg/kg mannitol and fluid restriction at a rate of 2 to 4 mL/kg/h during donor hepatectomy. In group B (blue box), an SVV of <10% was maintained by fluid management administered at a rate of 6 to 10 mL/kg/h, without diuretics, during surgery. Note that loss of red cell mass in group A was significantly lower than in group B. Colored bars indicate the mean, and error bars indicate the SD. SVV = stroke volume variation.

The amount of red cell mass loss was 145.4 ± 107.6 mL in group A compared with 307.9 ± 110.7 mL in group B (*P* < 0.001, Fig. 1). Postoperative outcomes are presented in Table 2. There were no significant differences between the 2 groups in serum creatinine, liver enzyme, or serum albumin levels through postoperative day 7. There were also no significant differences in incidences of acute kidney injury, abnormal chest radiographic findings, or duration of hospital stay between the 2 groups.

**TABLE 2:**
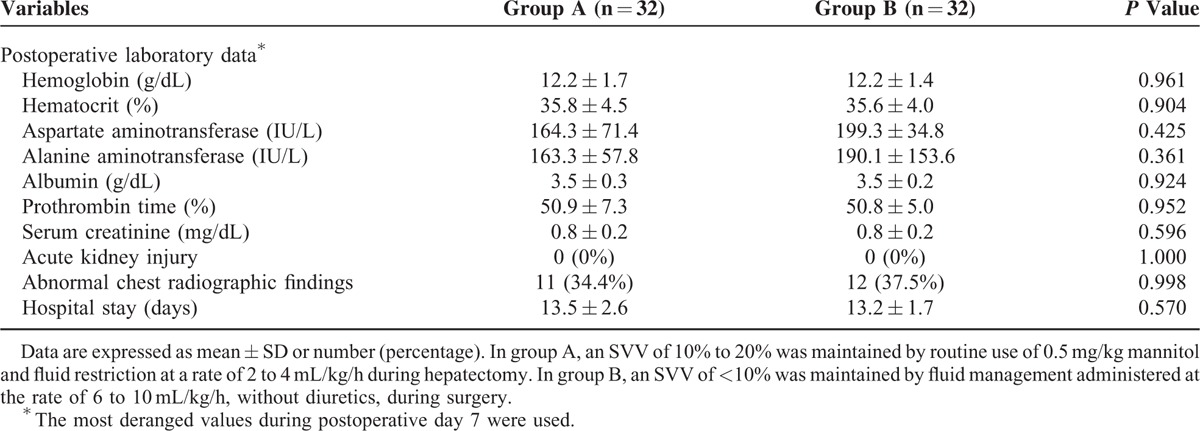
Postoperative Outcomes of 64 Living Liver Donors

## DISCUSSION

In our current study, we evaluated the effect of high SVV of 10% to 20% by mannitol administration and fluid restriction on intraoperative blood loss in living liver donors. We found that blood loss in group A (SVV maintained at 10–20% during donor hepatectomy) was significantly lower than in group B (SVV maintained <10% during donor hepatectomy). We also found that mannitol safely and effectively maintained a high SVV to reduce intraoperative blood loss.

SVV is a useful dynamic index that indicates fluid responsiveness and can be simply measured by arterial waveform analysis in mechanically ventilated patients.^19^ Although there are some clinical limitations in using a dynamic index,^20^ it has been reported that SVV is useful as a preload index and correlates well with CVP, which has been recommended as a conventional guide for fluid therapy during hepatic resection.^7,21^ As SVV predicts a preload dependent condition,^5^ maintaining a high SVV of 10% to 20% might imply a restricted intravascular volume during surgery, preventing vascular congestion of surgical areas and decreasing intraoperative blood loss. In our previous study, SVV showed a significant correlation with intraoperative blood loss during donor hepatectomy.^22^ SVV values also corresponded to CVP levels, and SVV levels of 18% to 21% were significantly correlated with CVP values of −1 to 1 mm Hg during hepatic resection.^7^ In addition, maintaining an SVV >18% resulted in a significantly lower estimated blood loss in patients who underwent hepatic resections under inferior vena cava and portal triad clamping.^21^ Moreover, our previous study also revealed that maintaining a high SVV of 10% to 20% can reduce intraoperative blood loss in donor right hepatectomy.^8^

Diuretics can be required to effectively maintain a high SVV of 10% to 20% during donor hepatectomy. In our previous study, we administered up to 40 mg of furosemide and possibly achieved an SVV of 10% to 20% during donor hepatectomy.^8^ In clinical practice, loop diuretics, particularly furosemide, can increase urine output and contribute to negative fluid balance.^23^ However, furosemide can also aggravate renal dysfunction in oliguric patients and lack effectiveness for improving renal function in many clinical situations.^10,11,24^ Although we found no significant changes in perioperative serum creatinine levels after administering small doses of furosemide during healthy living liver donor hepatectomy in our previous study,^8^ we believe that clinicians should consider the risks and benefits of furosemide during surgery. Alternative diuretics that are safe and effective and maintain a SVV of 10% to 20% during donor hepatectomy are required. Mannitol can induce osmotic diuresis, thereby helping prevent increased intravascular volume status. Moreover, mannitol has several advantages for renal function. Mannitol can cause renal vasodilation, maintain the renal filtration fraction, and maintain the renal oxygen balance between supply and demand.^14^ Also, mannitol has shown free radical scavenging activity in patients receiving liver resection with hepatic vascular exclusion.^13^ Although the benefits of mannitol on postoperative kidney and liver function are still controversial,^13,25^ mannitol can be effectively used as an osmotic diuretic to achieve the restricted intravascular volume status. In our present study, we found that mannitol safely and effectively maintained a high SVV of 10% to 20% to reduce blood loss during living donor hepatectomy.

We found in our present analyses that the high SVV method was effective in reducing blood loss during donor hepatectomy. Although the effectiveness of the conventional low CVP technique is the subject of debate,^26,27^ it is known to be beneficial in reducing blood loss during donor hepatectomy.^28^ However, central venous catheterization is reported to be associated with several catheter-related and procedure-related complications.^29^ Furthermore, central venous catheterization is not a simple procedure because it requires ultrasonography and perfectly sterile conditions.^29^ Hence, the high SVV method may be a simpler and safer method to reduce blood loss during donor hepatectomy.^8^

There were several limitations to our present study. First, we calculated intraoperative blood loss using the loss of red cell mass instead of conventional visual estimation using the sum of blood volume contained in the suction systems and gauzes. The visual estimation of intraoperative blood loss is thought to be inaccurate and may lead to underestimation.^30–32^ In our present analysis, we used patient hematocrit, an objective parameter, and a mathematical model to standardize blood loss estimation.^30^ Second, surgical techniques and the surgeon's experience are important determinants of intraoperative blood loss. However, the surgeons at our hospital have extensive experience with living donor liver transplantation,^33^ and only cases of right hepatectomy were included in our study series. Therefore, our results should have been only minimally influenced by surgical factors.

In conclusion, the maintenance of a high SVV of 10% to 20% is effective in reducing blood loss during living donor hepatectomy and is easily achievable through mannitol administration and fluid restriction. Our current findings provide a better understanding of the perioperative management techniques required to reduce intraoperative blood loss and maximize the safety of healthy liver donors.
